# Multifocal fixed drug eruption due to ornidazole

**DOI:** 10.1186/2045-7022-4-S3-P99

**Published:** 2014-07-18

**Authors:** Fevzi Demirel, Abdullah Baysan, Sait Yesillik, Ozgur Kartal, Mustafa Gulec, Ugur Musabak, Osman Sener

**Affiliations:** 1 Gulhane Military Medical Academy and Medical School, Division of Immunology and Allergic Diseases, Turkey

## Introduction

Ornidazole, a synthetic nitroimidazole derivative, is widely used in clinical practice. Ornidazol has low rate of side effects, but sometimes may cause significant allergic reactions such as fixed drug eruption (FDE). We describe a case of multifocal FDE caused by ornidazole.

## Case report

A 40-year old woman applied to the Immunology and Allergy clinic with pruritic erythematous lesions over the lateral sides of both arms and right hip within 30 minutes after the ingestion of ornizadole 500 mg tablet. She reported that five recurrent reactions emerged with the same drug on the same locations previously, and their severity was increased consecutively. She was prescribed ornidazole for trichomonal vaginitis and she never warned physicians about the previous drug reactions. In view of the personal history and physical examination a diagnosis of FDE due to ornidazole was diagnosed. After a month from the last reaction allergy tests including prick, intradermal and patch tests were performed with ornidazole and metranidazole. Skin tests with 1/100 W/vol and 1/10 W/vol concentrations and undiluted form of the two drugs were negative. The patch tests consisted of the same drug concentrations were applied to the lesion site on her left arm. Readings were done at 48 hour later and positive results were obtained with ornidazole but not with metranidazole. A placebo controlled oral challenge was performed with metranidazole and no reaction was observed. Following, she contunied to receive metranidazole without any problem.

## Conclusion

The lesionel patch test is more diagnostic than other allergy tests at recürrent fixed drug reactions. The patch test may be performed with the same drug solutions as performed on skin prick and intradermal tests without adding petrolatum. Although metronidazole and ornidazole are chemically related drugs, and metranidazole is known more susceptible to cause FDE, metranidazole may be an alternative drug to ornidazole like our case.

